# Ileal Varices Treated with Balloon-Occluded Retrograde Transvenous Obliteration

**DOI:** 10.4021/gr2009.04.1286

**Published:** 2009-03-20

**Authors:** Takahiro Sato, Katsu Yamazaki, Jouji Toyota, Yoshiyasu Karino, Takumi Ohmura, Jun Akaike

**Affiliations:** a Department of Gastroenterology, Sapporo Kosei General Hospital, Kita 3 Higashi 8, Chuo-ku, Sapporo 060-0033, Japan

**Keywords:** Balloon-occluded retrograde transvenous obliteration, Ectopic varices, Ileal varices, Interventional radiology, Portal hypertension

## Abstract

A 55-year-old man with hepatitis B virus antigen-positive liver cirrhosis was admitted to our hospital with anal bleeding. Colonoscopy revealed blood retention in the entire colon, but no bleeding lesion was found. Computed tomography images showed that vessels in the ileum were connected to the right testicular vein, and we suspected ileal varices to be the most probable cause of bleeding. We immediately performed double balloon enteroscopy, but failed to find any site of bleeding owing to the difficulty of fiberscope insertion with sever adhesion. Using a balloon catheter during retrograde transvenous venography, we found ileal varices communicating with the right testicular vein (efferent vein) with the superior mesenteric vein branch as the afferent vein of these varices. We performed balloon occluded retrograde transvenous obliteration by way of the efferent vein of the varices and have detected no further bleeding in this patient one year after treatment.

## Introduction

Ectopic varices that are not esophagogastric are located predominantly in the duodenum, jejunum, ileum, colon, rectum, or enterostomy stoma [1]. Bleeding from ileal varices, which is rare in patients with portal hypertension, is generally massive and life threatening. Diagnosis of ruptured ileal varices and control of bleeding are difficult.

Endoscopic injection sclerotherapy (EIS) is a standard procedure for treatment of esophageal varices [2]. More recently, endoscopic variceal ligation (EVL) has been applied widely in the treatment of esophageal varices [3]. Although balloon-occluded retrograde transvenous obliteration (B-RTO) is a new interventional modality for gastric fundic varices [4], a definitive treatment for bleeding ileal varices has not been established. Here we present a case of ruptured ileal varices controlled successfully using B-RTO.

### Case Report

A 55-year-old man with hepatitis B virus antigen-positive liver cirrhosis was admitted to our hospital in October 2007 with anal bleeding. At 36 years of age, he underwent an operation of the ileocecum to remove a benign colonic tumor. At 46 year of age (in 1998), he first received EIS for treatment of esophageal varices and then underwent EIS in 1999 and 2003 for recurrent esophageal varices.

At the time of admission, the patient’s blood pressure was 120/62 mmHg, pulse 98 beats/min and regular, and body temperature 36.5°C. He had anemic conjunctivae, but there was no scleral icterus. The abdomen was soft and flat, there were no obvious abdominal masses. Bleeding was massive and recurrent, and blood transfusions were required. The fibergastroscopic examination on admission revealed small, red color-negative esophageal varices. Laboratory findings were: red blood cell count 209 x 10^4^ /mm^3^ (normal:353 - 466 x 10^4^ /mm^3^), hemoglobin 5.6g/dL (10.6 - 14.4 g/dL), white blood cell count 6900/mm^3^ (3000 - 7800/mm^3^), platelet count 13.2 x 10^4^ /mm^3^ (13.8 - 30.9 x 10^4^ /mm^3^), serum albumin 2.6 g/mL (4.0 - 5.2 g/mL), total bilirubin 0.6 mg/mL (0.2 - 1.2 mg/mL), glutamic oxaliacetic transaminase 14 IU/L (8 - 38 IU/L), glutamic pyruvic transaminase 18 IU/L (4 - 44 IU/L), alkaline phosphatase 184 IU/L (104 - 338 IU/L), blood urea nitrogen 22.9 mg/dL (7.0 - 24.0 mg/dL), and creatine 0.9 mg/dL (0.4 - 0.9 mg/dL). Prothrombin time was 54% (90 -140%) and serological assays for markers of hepatitis B viruses were positive. All test results for tumor markers were within the normal range.

**Figure 1:**
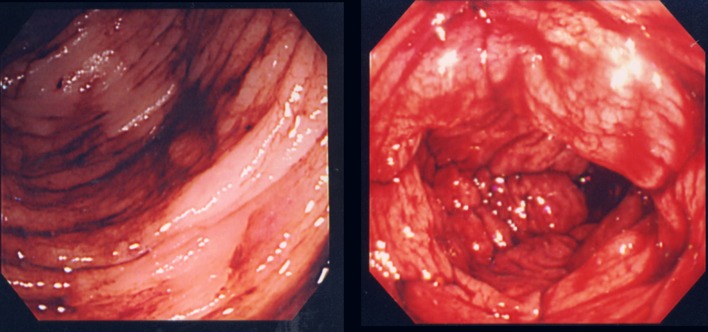
Colonoscopy revealed blood retention in the entire colon, but no bleeding lesion was detected.

**Figure 2:**
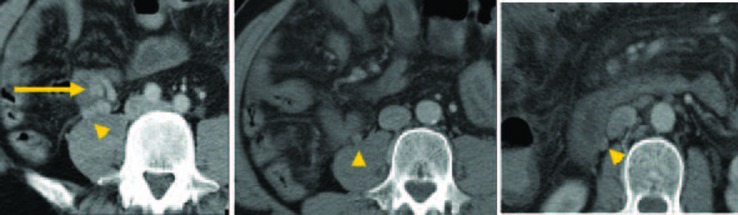
Computed tomography images of a vessel in the ileum (arrow) and its connection to the right testicular vein (arrowhead).

**Figure 3:**
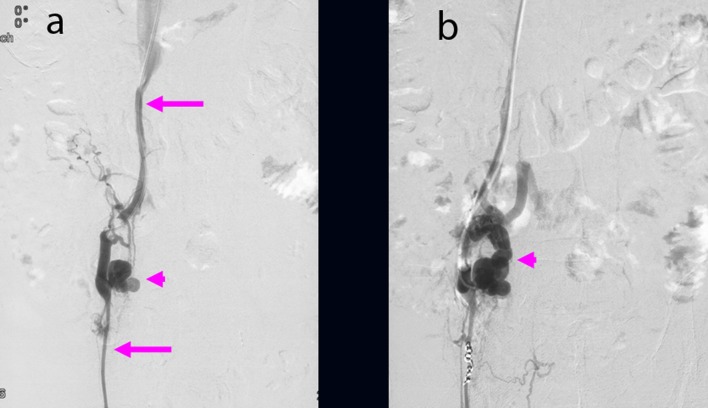
(a) Ileal varices (arrowhead) communicating with the right testicular vein (arrow) were found using retrograde transvenous venography. (b) Balloon occluded retrograde transvenous obliteration for ileal varices (arrowhead) was performed via the efferent vein of the varices.

Although colonoscopy revealed blood retention in the entire colon, no bleeding lesions were found (Fig. 1). Because computed tomography (CT) images of vessels in the ileum showed connection to vessels in the right testicular vein (Fig. 2), we suspected ileal varices to be the most probable cause of bleeding. We immediately performed double balloon enteroscopy, but failed to find any site of bleeding owing to the difficulty of fiberscope insertion with sever adhesion. Subsequent interventional radiology involving retrograde transvenous venography using balloon catheter from right subclavian vein revealed ileal varices communicating with the right testicular vein (efferent vein) (Fig. 3a). Angiography revealed that the superior mesenteric vein branch was the afferent vein for the ileal varices.

**Figure 4:**
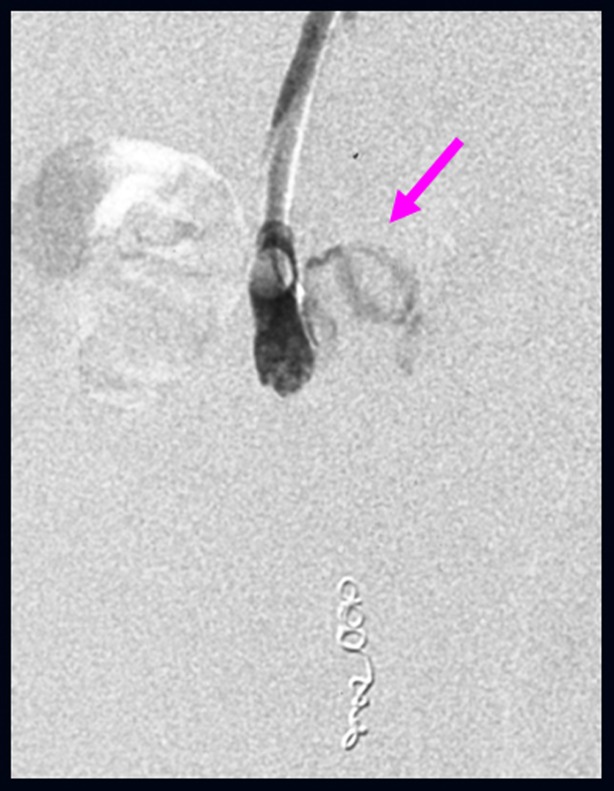
Following balloon occluded retrograde transvenous obliteration, both ileal varices (arrow) and the afferent vein were not visible by venography the next day.

**Figure 5:**
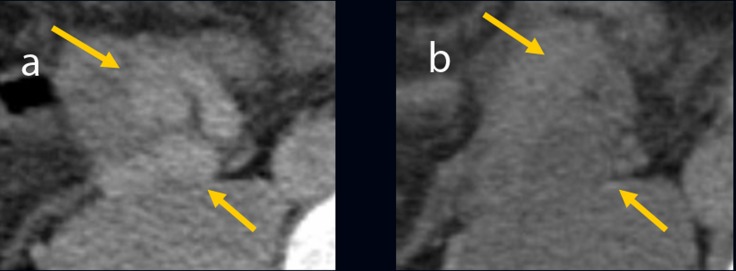
(a) Computed tomography image of ileal vessels before balloon occluded retrograde transvenous obliteration. (b) The ileal vessels are no longer visible following balloon occluded retrograde transvenous obliteration.

We performed B-RTO via the efferent vein of the varices. Because of the insufficient varicealography of ileal varices caused by rapid washout of the contrast medium, we performed microcoil embolization using 0.014-inch steel coils for the distal right testicular vein and injected 18 ml of 5% ethanolamine oleate (EO) containing iopamidol (Fig. 3b). There were no post-operative complications. After B-RTO, venography on the next day could not distinguish the ileal varices or the afferent vein (Fig. 4), and ileal vessel images before B-RTO disappeared after the treatment under CT (Fig. 5). One year after treatment, the patient had experienced no further bleeding.

## Discussion

Portal hypertension can involve either reopening of collapsed embryonic channels or a reversed in flow within existing adult veins [5]. Whereas esophagogastric varices are the most common complication in patients with portal hypertension, ectopic varices defined by large portosystemic venous collaterals occurring anywhere in gastrointestinal tract except in the esophagogastric region are less common and account for between 1% and 5% of all variceal bleeding [6, 7]. Several cases of bleeding ileal varices have been reported [8-16].

Ectopic varices have been reported to occur at numerous sites, including 18% in the jejunum or ileum, 17% in the duodenum, 14% in the colon, 8% in the rectum, and 9% in the peritoneum [1]. Most bleeding jejunal and ileal varices, generally detected previous intra-abdominal surgery, are serious due to the difficulty of early diagnosis. In our case, the patient's risk factors included portal hypertension due to liver cirrhosis, EIS for esophageal varices, and previous surgery. Collaterals formation within adhesions from a previous surgery is the usual mechanism for the development of ectopic varices [1]. Adhesions tend to bring the parietal surface of the viscera in contact with the abdominal wall, and portal hypertension results in the formation of varices below intestinal mucosa. Double balloon enteroscopy has gained worldwide acceptance as an endoscopic technique that can be used safely and effectively to provide complete examination of the small bowel, offer therapeutic intervention, and favorably affect clinical outcomes [17-19]. In this case, we immediately performed double balloon enteroscopy to find any site of bleeding, but failed to diagnose owing to the difficulty of fiberscope insertion with sever adhesion.

Surgical approaches such as segmental resection and ligation generally control bleeding from ileal varices successfully [12, 13, 20, 21]. In patients with poor condition, interventional radiologic treatment such as insertion of a transjugular intrahepatic portosystemic shunt (TIPS) for ileal varices have been performed as a non-operative treatment option [7, 14, 16]. Although TIPS is a relatively safe and effective means of decompressing the portal pressure, it may not prove effective in patients with severe liver atrophy or complications such as encephalopathy and cerebral embolization. Because B-RTO can obliterate not only varices but also the afferent veins and efferent veins, it is practical for treating ileal varices [22], as described here. In the future, interventional radiologic treatments such as B-RTO may also be applied as therapy for patients in poor condition.

In summary, ileal varices should be considered in patients that have undergone previous surgery and display lower gastrointestinal bleeding and portal hypertension as well as endoscopic findings that do not reveal any bleeding point.
